# Identification of oxidative stress-related diagnostic markers and immune infiltration features for idiopathic pulmonary fibrosis by bibliometrics and bioinformatics

**DOI:** 10.3389/fmed.2024.1356825

**Published:** 2024-08-06

**Authors:** Chang Li, Qing An, Yi Jin, Zefei Jiang, Meihe Li, Xiaoling Wu, Huimin Dang

**Affiliations:** ^1^Department of Traditional Chinese Medicine, The Second Affiliated Hospital of Xi’an Jiaotong University, Xi’an, China; ^2^Graduate School, Shaanxi University of Chinese Medicine, Xianyang, China; ^3^Acupuncture and Tuina School, Chengdu University of Traditional Chinese Medicine, Chengdu, China; ^4^Department of Renal Transplantation, The First Affiliated Hospital of Xi’an Jiaotong University, Xi’an, China; ^5^Department of Obstetrics and Gynecology, The Second Affiliated Hospital of Xi’an Jiaotong University, Xi’an, China

**Keywords:** oxidative stress, immune infiltration, idiopathic pulmonary fibrosis, bibliometrics, bioinformatics

## Abstract

Idiopathic pulmonary fibrosis (IPF) garners considerable attention due to its high fatality rate and profound impact on quality of life. Our study conducts a comprehensive literature review on IPF using bibliometric analysis to explore existing hot research topics, and identifies novel diagnostic and therapeutic targets for IPF using bioinformatics analysis. Publications related to IPF from 2013 to 2023 were searched on the Web of Science Core Collection (WoSCC) database. Data analysis and visualization were conducted using CiteSpace and VOSviewer software primarily. The gene expression profiles GSE24206 and GSE53845 were employed as the training dataset. The GSE110147 dataset was employed as the validation dataset. We identified differentially expressed genes (DEGs) and differentially expressed genes related to oxidative stress (DEOSGs) between IPF and normal samples. Then, we conducted Gene Ontology (GO) enrichment and Kyoto Encyclopedia of Genes and Genomes (KEGG) pathway enrichment analysis. The hub genes were screened by protein–protein interaction (PPI) networks and machine learning algorithms. The CIBERSORT was used to analyze the immune infiltration of 22 kinds of immune cells. Finally, we conducted the expression and validation of hub genes. The diagnostic efficacy of hub genes was evaluated by employing Receiver Operating Characteristic (ROC) curves and the associations between hub genes and immune cells were analyzed. A total of 6,500 articles were identified, and the annual number of articles exhibited an upward trend. The United States emerged as the leading contributor in terms of publication count, institutional affiliations, highly cited articles, and prolific authorship. According to co-occurrence analysis, oxidative stress and inflammation are hot topics in IPF research. A total of 1,140 DEGs were identified, and 72 genes were classified as DEOSGs. By employing PPI network analysis and machine learning algorithms, PON2 and TLR4 were identified as hub genes. A total of 10 immune cells exhibited significant differences between IPF and normal samples. PON2 and TLR4, as oxidative stress-related genes, not only exhibit high diagnostic efficacy but also show close associations with immune cells. In summary, our study highlights oxidative stress and inflammation are hot topics in IPF research. Oxidative stress and immune cells play a vital role in the pathogenesis of IPF. Our findings suggest the potential of PON2 and TLR4 as novel diagnostic and therapeutic targets for IPF.

## 1 Introduction

Idiopathic pulmonary fibrosis (IPF) is a chronic, progressive interstitial lung disease associated with irreversible lung fibrosis (1–4). Despite being relatively low prevalence on a global scale, it garners considerable attention due to its high fatality rate and profound impact on quality of life (5–9). In the study conducted by Maher et al., the incidence rate of IPF ranged from 0.35 to 1.30 per 10,000 individuals in the Asia-Pacific region, 0.09–0.49 in Europe, and 0.75–0.93 in North America (10). Collard et al. (8) demonstrated that individuals with IPF exhibited a 134% elevated likelihood of hospitalization and 134% greater total medical expenses compared to control individuals. Given the devastating nature of IPF, it is crucial to explore existing hot research topics, undertake an in-depth investigation of pathogenesis, and identify potential diagnostic and therapeutic biomarkers.

Significant advancements in IPF have been achieved over the last few decades, particularly in terms of pathological responses and molecular mechanisms (6, 11–16). Initially, inflammation was considered to play a crucial role in the pathogenesis of IPF (4, 17, 18). However, recent findings have shed light on the critical involvement of oxidative stress in IPF (15, 19–24). Due to exposure to elevated levels of oxygen, the lungs are particularly susceptible to oxidative stress (22, 25, 26). Hydrogen peroxide and reactive oxygen species (ROS) generated by alveolar inflammatory cells usually serve as markers for oxidative stress (27–29). The study conducted by Daniil et al. (30) has demonstrated significantly elevated levels of total hydrogen peroxide in the serum of IPF individuals. Moreover, the severity of IPF is positively associated with the concentration of hydrogen peroxide in the serum. Additionally, ROS are regarded as crucial for IPF because of the ability to impact key mediators such as transforming growth factor β (TGF-β) in the pathogenesis of IPF (27, 31–33).

In recent times, there has been a growing interest in the application of bibliometrics and bioinformatics analysis in the healthcare domain (34–36). Our study conducts a comprehensive literature review on IPF using bibliometric analysis, especially focusing on oxidative stress and inflammation. Additionally, we aim to confirm or identify novel diagnostic and therapeutic targets for IPF using bioinformatics analysis.

## 2 Materials and methods

### 2.1 Data source and search strategy of bibliometrics

**Figure 1:**
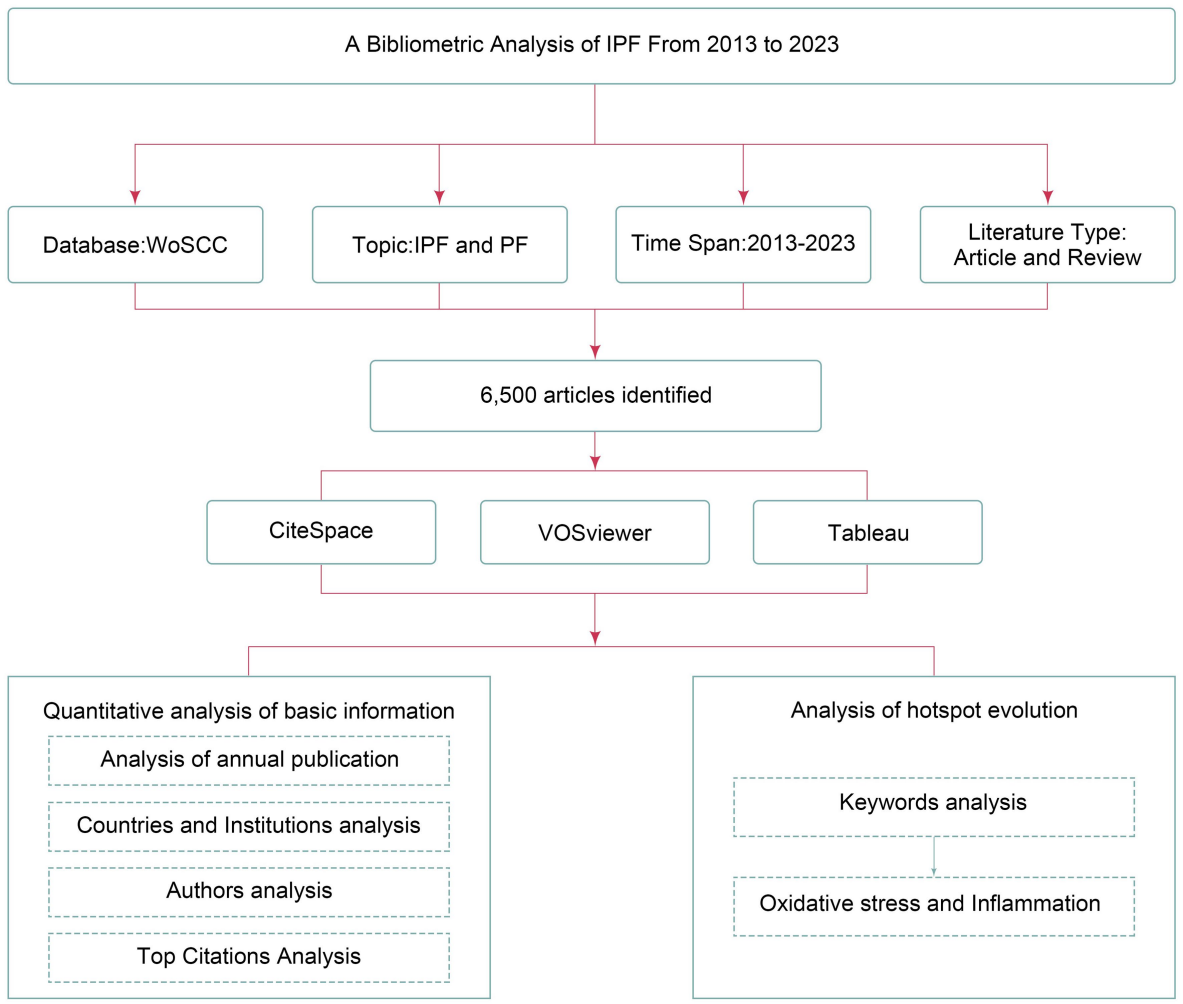
Flowchart for bibliometrics analysis.

As shown in Figure 1, the bibliometric data were obtained from the Web of Science Core Collection (WoSCC), a comprehensive database encompassing various disciplines such as natural science, medicine, and social science. The search strategy is [TS = (“Idiopathic Pulmonary Fibrosis”) OR TS = (“Pulmonary Fibrosis”) OR TS = (“IPF”) OR TS = (“PF”)]. The search was carried out over a period spanning from January 2013 to November 2023, and the outcomes were acquired on December 1, 2023, within a single day.

### 2.2 Inclusion and exclusion criteria

Our study encompassed published articles and reviews written in English, while excluding case reports, abstracts, retracted articles, and meeting reports. The titles and abstracts underwent independent evaluation by two researchers, with any disagreements being resolved through arbitration by a third author.

### 2.3 Data extraction and analysis of bibliometrics

The following indicators were recorded: the number of articles published, article types, publication years and countries, institutions of publication, authors, keywords, and total citations. Data analysis and visualization were conducted using CiteSpace and VOSviewer software primarily.

### 2.4 Data collection of bioinformatics analysis

**Figure 2:**
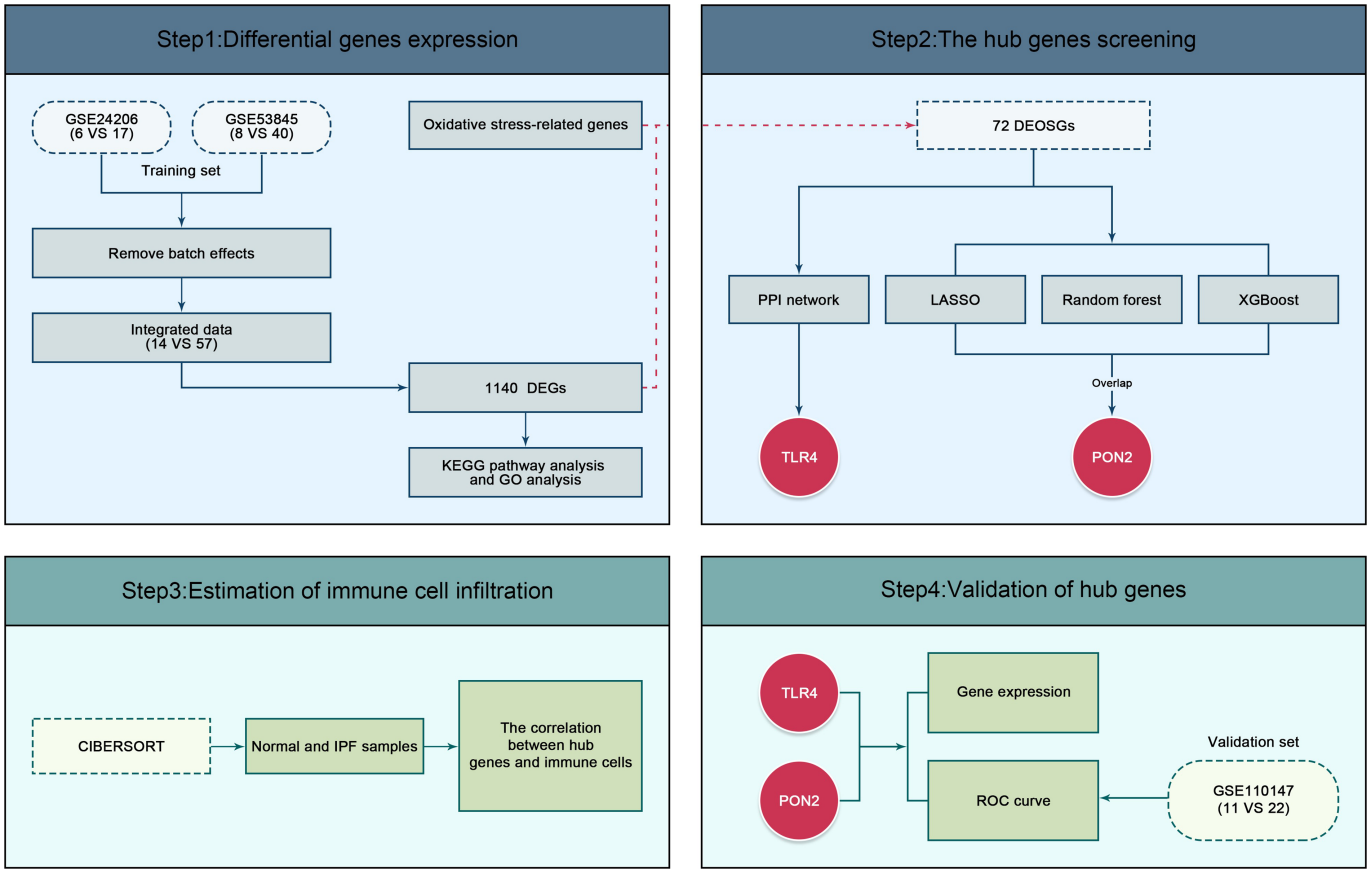
Flowchart for bioinformatics analysis.

As shown in Figure 2, the gene expression profiles GSE24206 and GSE53845 were obtained from the National Center of Biotechnology Information (NCBI) Gene Expression Omnibus (GEO) database and employed as the training dataset. The GSE24206 dataset was based on the GPL570 Platform, respectively, the GSE53845 dataset was based on the GPL6480 Platform. The GSE110147 dataset, based on the GPL6244 Platform was employed as the validation dataset. A total of 104 samples were selected, comprising 57 IPF and 14 normal samples in the training dataset, and 22 IPF and 11 normal samples in the validation dataset. The gene sets associated with oxidative stress were acquired from MSigDB.

### 2.5 Identification of differentially expressed genes and differentially expressed genes related to oxidative stress

R/Bioconductor was employed for preprocessing and statistical analysis. Specifically, the R packages “limma” and “sva” were utilized to mitigate batch effects across datasets and identify DEGs between IPF and normal samples. DEGs were screened based on the cut-off criteria of a *p* value <0.05 and |log2 fold change (FC) | >1.5. To visualize the results, volcano plots and heat maps were generated using the “ggplot2” and “heatmap” packages. Additionally, DEOSGs were obtained by intersecting the DEGs with gene sets associated with oxidative stress.

### 2.6 Gene ontology enrichment and Kyoto encyclopedia of genes and genomes pathway enrichment analysis

In this study, we conducted GO enrichment analysis and KEGG pathway enrichment analysis using the “clusterProfiler” package. The results were visualized using the “ggplot2” package, and a significance level of *p* < 0.05 was used as the cut-off criteria.

### 2.7 Identification of hub genes using protein–protein interaction network and machine learning algorithms

The PPI network was constructed using the STRING database.^1^ The identification of central genes was performed using the cytoHubba plug-in of Cytoscape software.

On the other hand, three machine learning algorithms, including Least Absolute Shrinkage and Selection Operator (LASSO) logistic regression, Extreme gradient boosting (XGBoost) algorithm, and Random Forest (RF) algorithm were employed to identify diagnostic biomarkers. The “glmnet” package, “xgboost” package, and “randomForest” package were used to implement the machine learning algorithms. The overlapping sets of diagnostic biomarkers obtained from the three machine learning algorithms were visualized using Venn diagrams. Ultimately, the hub genes were determined by integrating the genes obtained through the PPI network and machine learning algorithms.

### 2.8 Immune infiltration analysis

The CIBERSORT algorithm was used to assess the immune infiltration of 22 immune cells. Visualization of the results was conducted using the R packages “corplot,” “vioplot,” and “ggplot2.”

### 2.9 Expression and validation of hub genes

The expression levels of hub genes were visualized by violin plots. The diagnostic efficacy of hub genes was evaluated by employing Receiver Operating Characteristic (ROC) curves and visualized using the “pROC” package. Furthermore, the associations between hub genes and immune cells were computed and represented using a heatmap.

## 3 Results

**Figure 3:**
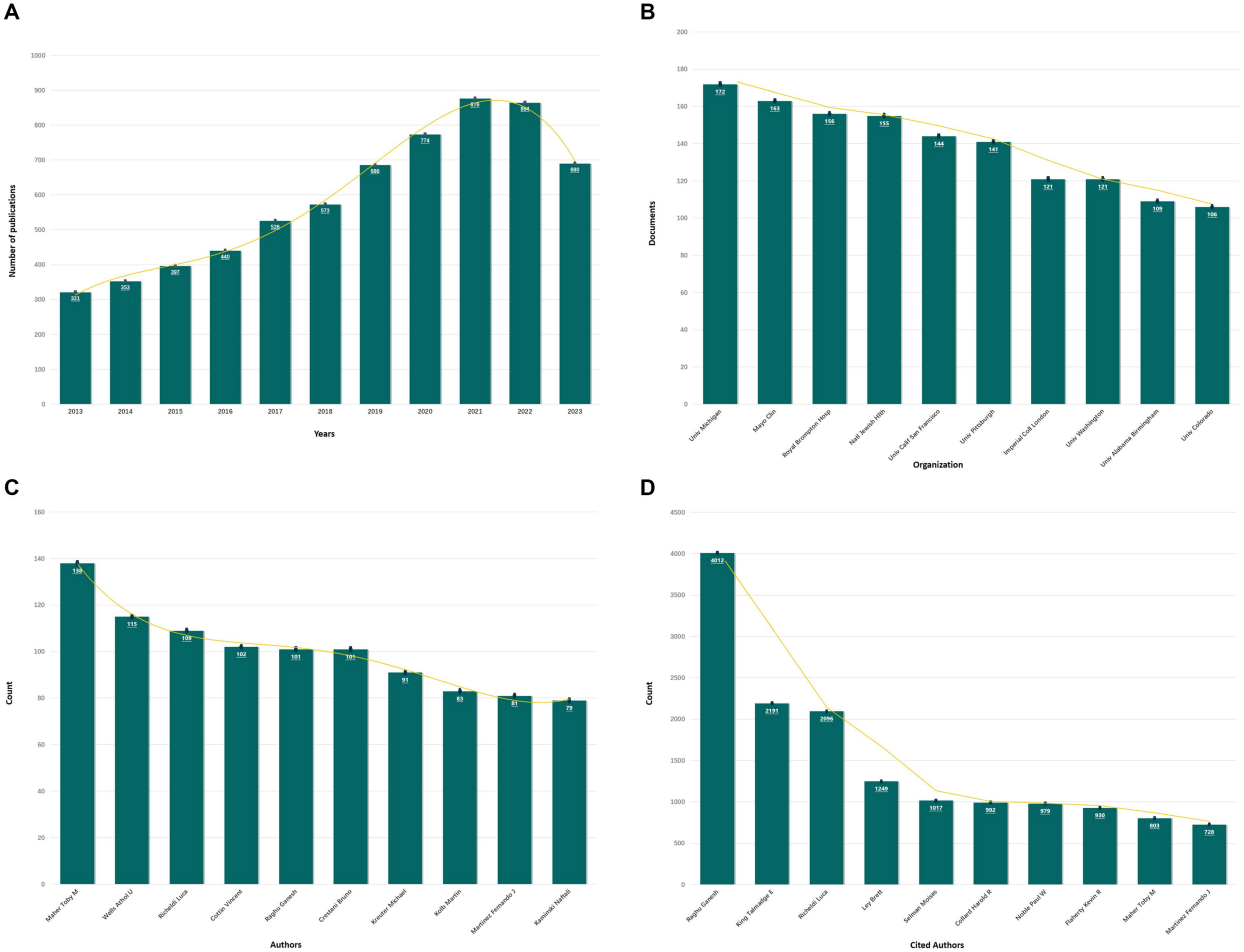
The results of bioinformatics analysis. **(A)** Annual publication volume of IPF from 2013 to 2023. **(B)** The top 10 institutions with the highest number of articles. **(C)** The top 10 authors with the highest number of articles. **(D)** The top 10 most cited authors.

As shown in Figure 3, a total of 6,500 articles were identified, including 5,291 original articles (81.4%) and 1,209 review articles (18.6%). The annual number of articles exhibited an upward trend, reaching a peak of 876 in 2021.

### 3.1 Leading countries and institutions

A total of 87 countries and 5,663 institutions made contributions to the publications on IPF. Figure 3 illustrates that the United States had the highest number of articles (1,654 articles, 25.45%), followed by China (1,160 articles, 17.85%), Japan (616 articles, 9.48%), Italy (386 articles, 5.94%), and Germany (292 articles, 4.49%). Among the institutions, the University of Michigan had the highest number of articles, followed by the Mayo Clinic, Royal Brompton Hospital, National Jewish Health, and the University of California, San Francisco.

### 3.2 Leading authors and the most cited paper

A total of 28,160 authors participated in the publication of the articles. As depicted in Figure 3, Maher from the University of London Imperial College of Science emerged as the most prolific author (138 articles, 2.12%). Among the top six productive authors, each contributed over 100 articles. Additionally, the following authors garnered the highest number of citations: Raghu (*n* = 4,012), King (*n* = 2,191), Richeldi (*n* = 2096), Ley (*n* = 1,249), and Selman (*n* = 1,017). The article with the highest citation count, titled “Diagnosis of Idiopathic Pulmonary Fibrosis: An Official ATS/ERS/JRS/ALAT Clinical Practice Guideline,” was authored by Raghu and published in the American Journal of Respiratory and Critical Care Medicine in 2018, and the total number of citations was 952.

### 3.3 Analysis of keywords

**Figure 4:**
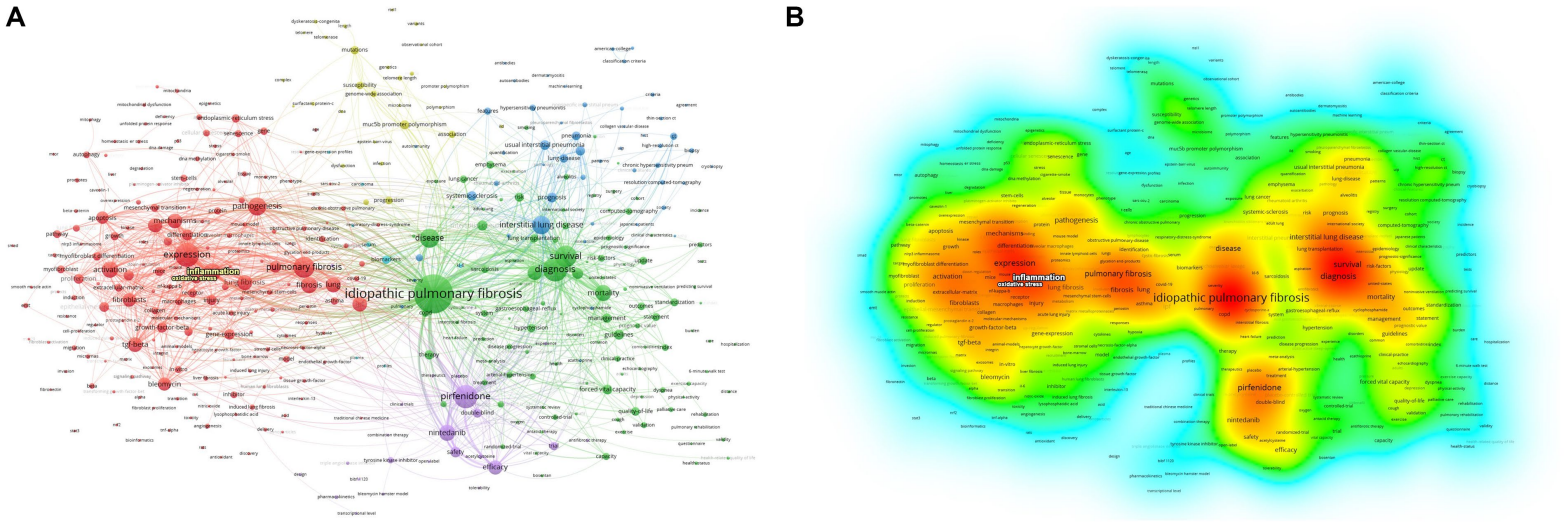
Network of related keywords. The size of each node indicates the frequency of the keywords, whereas the node labels show the keywords. **(A)** The cluster analysis of keywords. **(B)** The visualization of keywords density.

A total of 416 keywords were identified, with 40 of them surpassing the threshold of 200 occurrences. Notably, “idiopathic pulmonary fibrosis,” “diagnosis,” and “expression” emerged as the top three keywords with the highest frequency. As depicted in Figure 4, the keywords were effectively classified into six distinct clusters. Cluster 1 characterized by red consisted of a total of 193 keywords, with “oxidative stress “and “inflammation” being the terms that appeared more frequently. The frequency of occurrence and link strength of “oxidative stress” were found to be 241 and 1,489, respectively, and those of “inflammation” were 536 and 3,301.

### 3.4 Identification of DEGs and functional enrichment analysis

**Figure 5:**
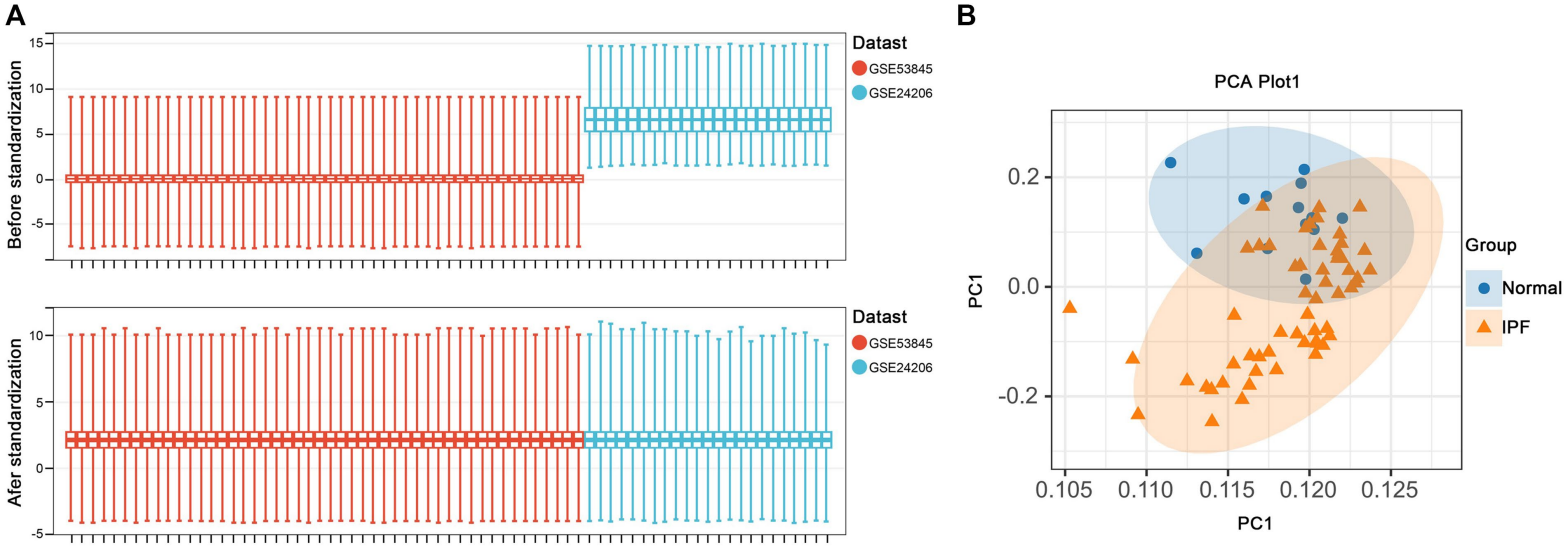
Data preprocessing. **(A)** Boxplots of gene expressions before and after standardization for selected GEO datasets. **(B)** PCA analysis demonstrates clustering in standardized samples.

**Figure 6:**
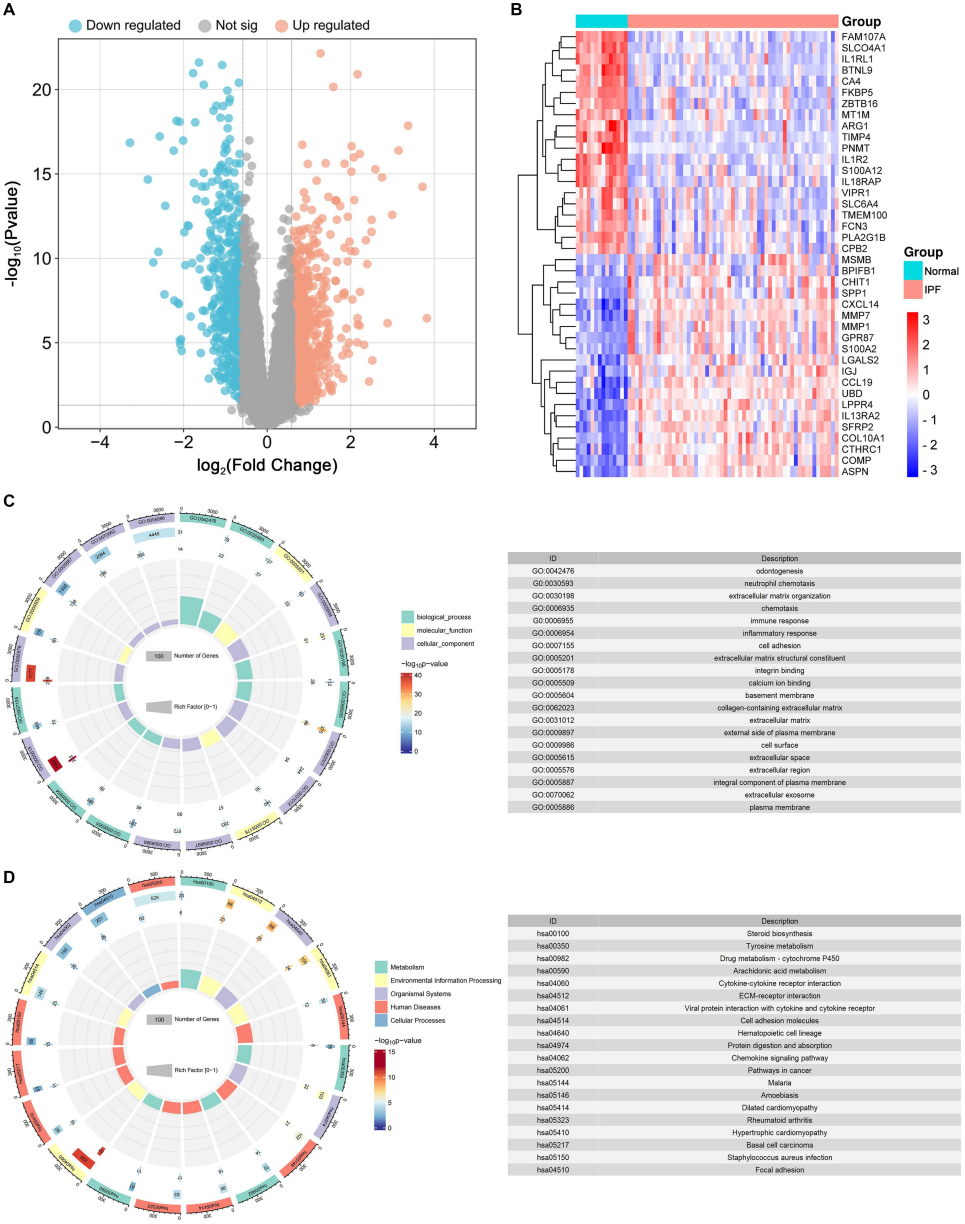
Identification of differentially expressed genes (DEGs) and functional enrichment analysis. **(A)** The volcano plot of the DEGs. **(B)** The heat map of the top 20 upregulated and top 20 downregulated DEGs. **(C)** The results of GO enrichment for DEGs. **(D)**The results of Kyoto Encyclopedia of Genes and Genomes (KEGG) enrichment for DEGs.

As depicted in Figures 5, 6, a total of 1,140 DEGs were identified, consisting of 616 upregulated DEGs and 524 downregulated DEGs. GO enrichment analysis indicated that DEGs were mainly enriched in odontogenesis, neutrophil chemotaxis, and extracellular matrix organization. The KEGG pathway enrichment analysis revealed a significant enrichment of DEGs in steroid biosynthesis, tyrosine metabolism, and drug metabolism-cytochrome P450.

### 3.5 PPI network analysis and machine learning of DEOSGs

**Figure 7:**
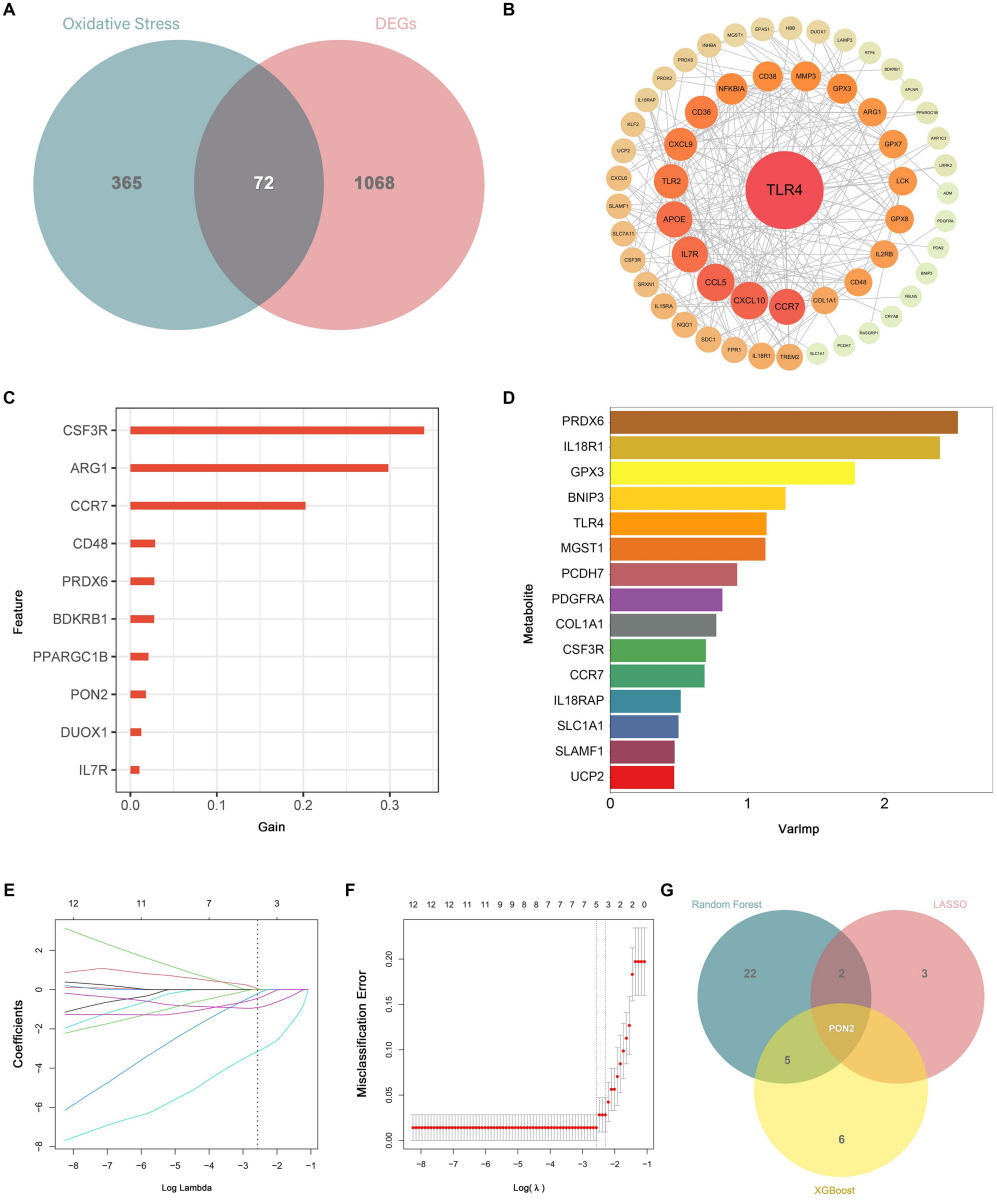
Identification of differentially expressed genes related to oxidative stress (DEOSGs) and hub genes. **(A)** Venn diagram showed the intersection of DEGs and oxidative stress-related genes. **(B)** TLR4 was identified as the central gene in the PPI network. **(C)** Diagnostic biomarkers were determined based on the XGboost algorithm. **(D)** Diagnostic biomarkers were determined based on the RF algorithm. **(E,F)** Diagnostic biomarkers were determined based on the LASSO algorithm. **(G)** Venn diagram showed the intersection of diagnostic markers obtained by the three machine algorithms.

By intersecting the DEGs with oxidative stress-related genes, a total of 72 DEOSGs were obtained. The PPI network contained 72 nodes and 233 edges, and the average node degree was 6.47. As shown in Figure 7, TLR4 was identified as the central gene in the network. Additionally, six diagnostic biomarkers were determined using the LASSO method, 12 diagnostic biomarkers were determined using the XGboost algorithm, and 30 diagnostic biomarkers were determined using the RF algorithm. After the intersection, only PON2 was identified as a diagnostic biomarker. Ultimately, TLR4 and PON2 were chosen as hub genes.

### 3.6 Immune infiltration analysis

**Figure 8:**
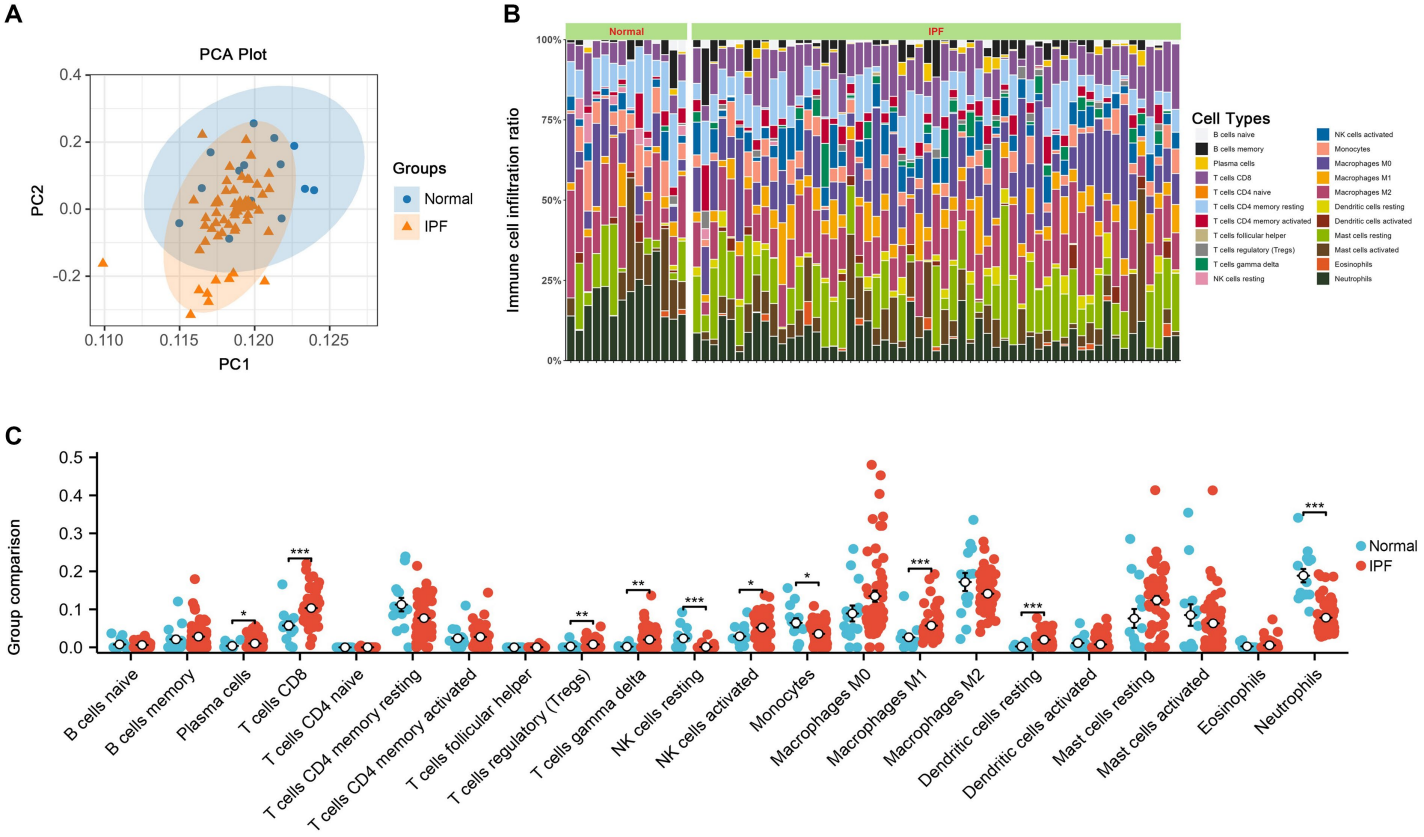
The results of immune infiltration. **(A)** PCA analysis was performed to classify immune cells between IPF and normal samples. **(B)** Immune cell content stacking plot between IPF and normal samples. **(C)** Immune cell content histogram.

As shown in Figure 8, a total of 10 immune cell types exhibited significant differences between IPF and normal samples, including Plasma cells, T cells CD8, T cells regulatory, T cells gamma delta, NK cells resting, NK cells activated, Monocytes, Macrophages M1, Dendritic cells resting, and Neutrophils.

### 3.7 Expression and validation of hub genes

**Figure 9:**
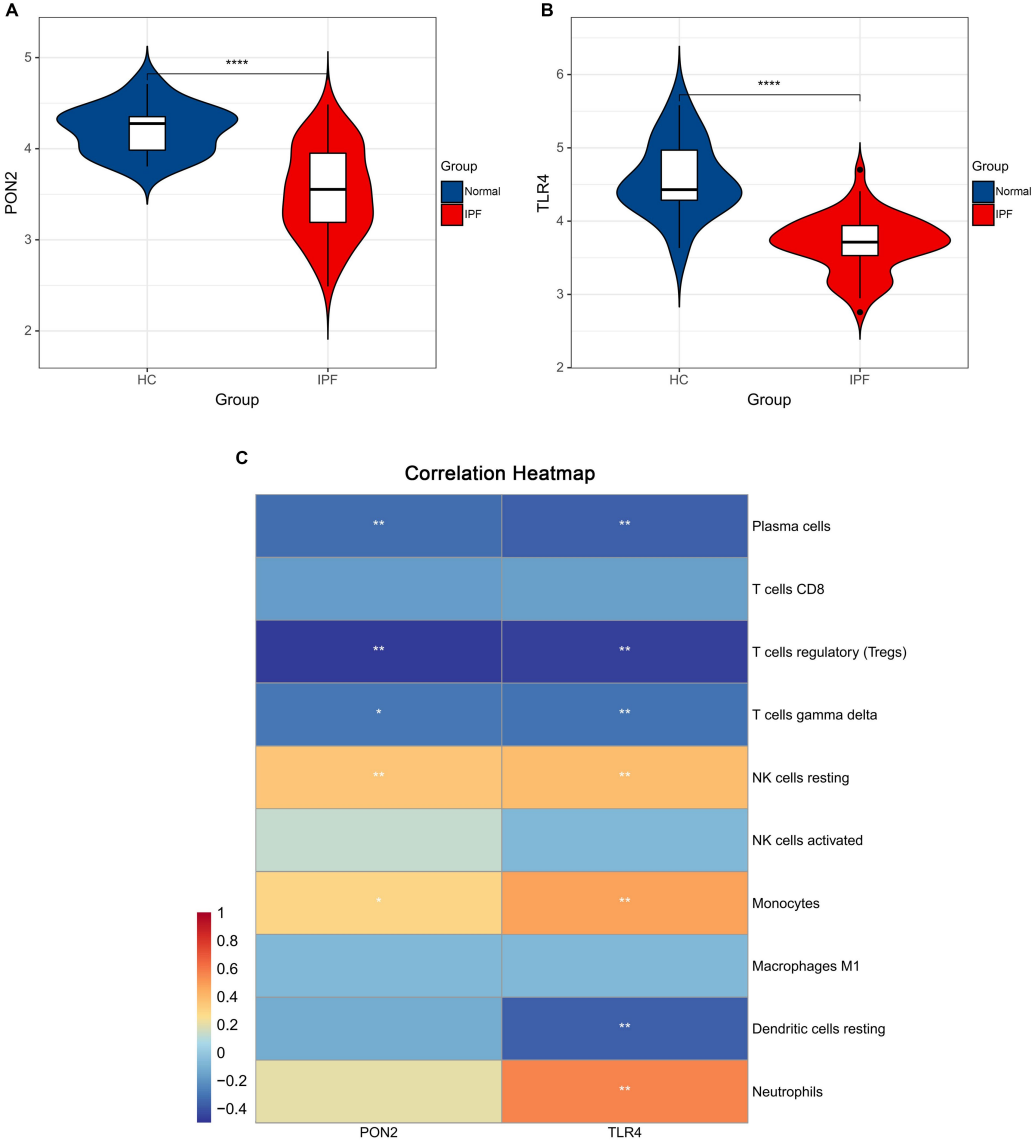
The expression levels of hub genes and the associations between hub genes and immune cells. **(A)** Violin plots exhibiting the expression level of PON2 in IPF and normal samples. **(B)** Violin plots exhibiting the expression level of TLR4 in IPF and normal samples. **(C)** The associations between hub genes and immune cells.

**Figure 10:**
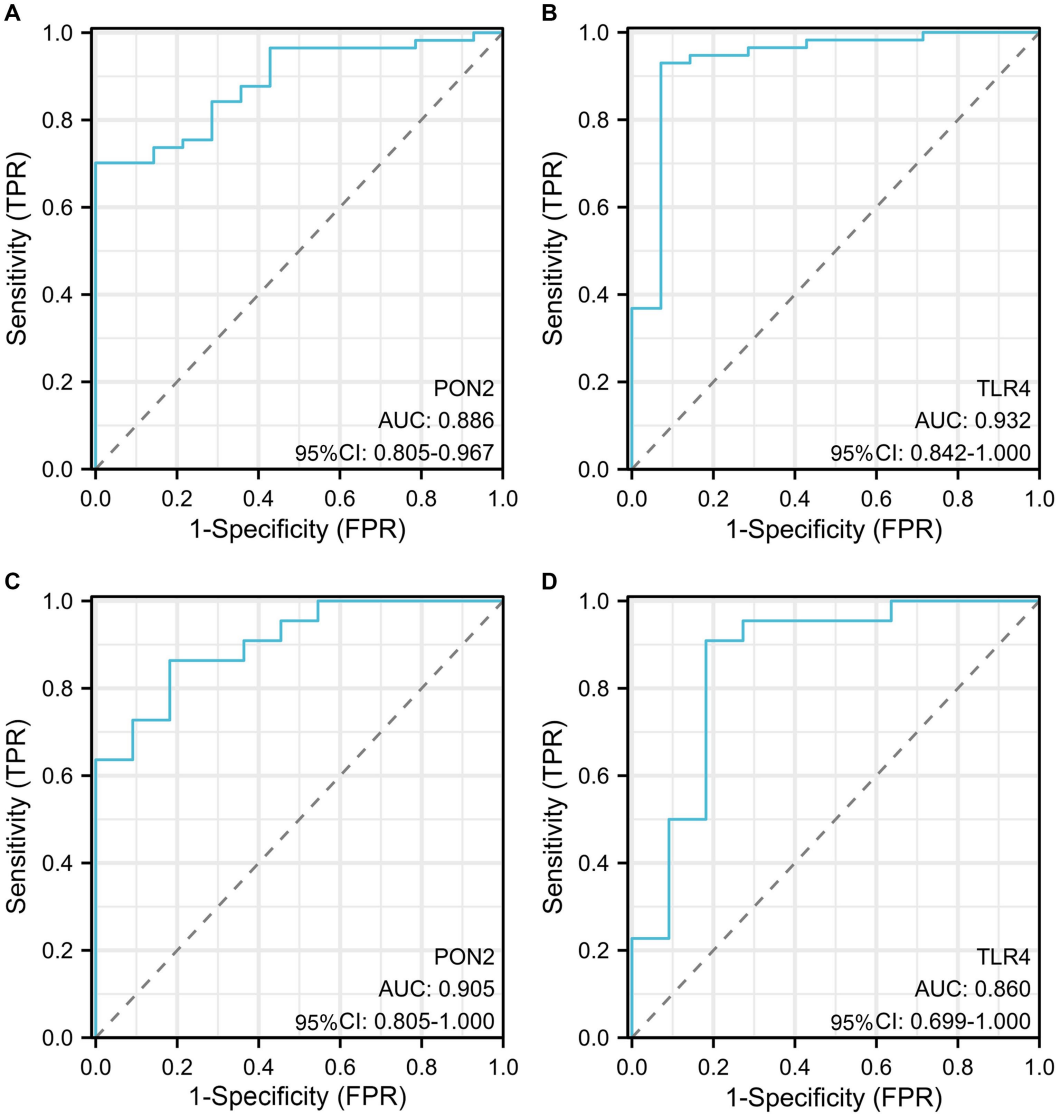
The diagnostic efficacy of hub genes. **(A)** The AUC value of PON2 in the training dataset. **(B)** The AUC value of TLR4 in the training dataset. **(C)** The AUC value of PON2 in the validation dataset. **(D)** The AUC value of TLR4 in the validation dataset.

As shown in Figures 9, 10, lower expression levels of PON2 and TLR4 in IPF samples compared to the normal sample were observed.

Significant negative associations between PON2 and Plasma cells, T cells regulatory, and T cells gamma delta, as well as positive associations between PON2 and NK cells resting, and Monocytes were observed. Significant negative associations between TLR4 and Plasma cells, T cells regulatory, T cells gamma delta, and Dendritic cells resting, as well as positive associations between TLR4 and NK cells resting, Monocytes, and Neutrophils were observed.

In the training dataset, the AUC value of PON2 was 0.886 (95% CI, 0.805–0.967), AUC value of TLR4 was 0.932 (95% CI, 0.842–1.000). In the validation dataset, the AUC value of PON2 was 0.905 (95% CI, 0.805–1.000), AUC value of TLR4 was 0. 860 (95% CI, 0. 699–1.000).

## 4 Discussion

In our study, a total of 1,140 DEGs were identified, and 72 genes were classified as DEOSGs. By employing PPI network analysis and machine learning algorithms, PON2 and TLR4 were identified as hub genes. Both of the two genes not only exhibit high diagnostic efficacy but also show close associations with immune cells.

Increasing research has confirmed the anti-oxidative stress effects of PON2 (37–39). Meilin et al. pointed out that PON2 plays a significant role in protecting against macrophage oxidative stress via reducing NADPH-oxidase activity (40). According to the research conducted by Sulaiman et al., PON2 demonstrates a protective effect against acute myocardial ischemia–reperfusion injury (IRI) by mitigating mitochondrial dysfunction and oxidative stress in cardiomyocytes (41). Although more and more diseases have been confirmed to be closely associated with PON2, there are few studies on its role in IPF (42–46). Our research proposes that PON2 could serve as a novel target for IPF investigations. It is not only an oxidative stress-related gene with significant diagnostic efficacy, but it also exhibits strong associations with immune cells in IPF.

The role of TLR4 in the development of IPF has attracted wide attention in recent years (47–50). The study conducted by Liang et al. highlights the importance of TLR4 and endogenous matrix glycosaminoglycan HA in limiting fibrosis after lung injury (49). Yang et al. pointed out that inhibition of TLR4 contributes to the promotion of an immunosuppressive tissue microenvironment, reduction in autophagy-associated collagen degradation, and mitigation of cell death in fibrotic lung tissues, which exacerbates bleomycin-induced pulmonary inflammation, fibrosis, dysfunction, and animal death. Furthermore, TLR4 activation expedites the resolution of acute inflammation, effectively reverses established pulmonary fibrosis, enhances lung function, and rescues mice from mortality (47). Our study demonstrated a lower expression level of TLR4 in the IPF samples and close associations with immune cells. The results suggest that the role of TLR4 in IPF is intricate and warrants further investigation.

Our study employed bibliometric methods to conduct an objective and quantitative analysis of 6,500 articles about IPF, which indicated a substantial increase in the number of articles from 2013 to 2023. The prevalence of original articles, accounting for 81.4%, indicated that IPF is a burgeoning and dynamic field. Meanwhile, the United States emerged as the leading contributor in terms of publication count, institutional affiliations, highly cited articles, and prolific authorship, underscoring its significant role and widespread recognition in this field. Additionally, according to co-occurrence analysis, oxidative stress and inflammation are hot topics in IPF research.

Recent research has indicated that oxidative stress may a vital role in the pathogenesis of IPF (22, 26–32). A study conducted by Beeh et al. (51) confirmed that IPF individuals exhibited lower levels of plasma and sputum glutathione (GSH), which are exceptional antioxidant small molecules, compared to healthy individuals. Yasuo et al. (52) pointed out that serum oxidative stress levels increased in IPF individuals as the disease progressed. Some studies have suggested that targeting Mitogen-activated protein kinase phosphatase-5 (MKP-5), which is associated with oxidative stress, seems to be beneficial for the treatment of IPF. Zhao et al. (53) pointed out that MKP-5 overexpression suppressed oxidative stress induced by palmitic acid (PA) by improving autophagic signaling. Xylourgidis et al. (54) demonstrated that MKP-5 expression was increased in IPF-derived lung fibroblasts but not in whole IPF lungs, implying that MKP-5 inhibition serve as a therapeutic target for IPF. Besides, depression, which is prevalent in IPF, is directly linked to oxidative stress. In the study conducted by Tzouvelekis et al. (55), 42.9% of IPF patients presented with depressive symptoms scoring ≥14, and these depressive symptoms were significantly associated with IPF disease severity. Indeed, the overproduction of reactive oxygen species and the lack of an effective antioxidant response in depressive states result in inflammation, neurodegeneration, and neuronal death (56). This highlights the complex interplay between oxidative stress and various comorbidities in IPF. In our study, we identified 72 DEOSGs and two hub genes. We analyzed the expression levels of the hub genes and explored their diagnostic efficacy.

Inflammation and immune dysregulation play a vital role in the IPF (29, 40, 42, 43). Hou et al. (57) observed a significant increase in peripheral blood and bronchoalveolar lavage (BAL) samples from IPF patients. Wei et al. (58) demonstrated an increased percentage of CD8+ T cells in the T cell subpopulation in the lungs of IPF patients, with these cells showing enriched fibrotic signaling pathways. Additionally, Daniil et al. (59) pointed out that the augmentation of CD8+ T cells in lung biopsies of IPF patients was associated with reduced total lung capacity and forced vital capacity. Papiris et al. (60) demonstrated that the CD8+ TLs correlated positively with the MRC grade and negatively with residual volume (RV), the neutrophils correlated positively with the MRC grade and negatively with the diffusing capacity for carbon monoxide (DLCO), PaO2, and PaCO2. Neutrophils contribute to the pathogenesis of IPF by producing cytokines and chemokines, responding to tissue injury, regulating extracellular matrix turnover, and generating neutrophil extracellular traps. These activities collectively result in fibroblast activation and extracellular matrix accumulation, thereby advancing fibrotic processes (61). Galati et al. observed that the percentage and absolute number of NK cells were significantly reduced in IPF patients, which aligns with our findings (62). In our study, seven immune cells were upregulated, including T cells regulatory and CD8+ T cells, and three immune cells were downregulated, including neutrophils and NK cells. The results underscore the critical role of immune cells in the progression of IPF, also emphasizing the necessity for further research to elucidate the intricate interactions among these cellular components.

Several limitations should be considered in our study. First, we only collected articles from the WoSCC database, and not checking other databases might result in the omission of crucial viewpoints in the IPF field. Second, the datasets used for bioinformatics were obtained from a publicly available database, lacking information on key clinical features such as sex, age, and therapeutic effects. Furthermore, the findings of our analysis require confirmation through *in vitro*, *in vivo*, and clinical trial studies.

## 5 Conclusion

In summary, our study highlights oxidative stress and inflammation are hot topics in IPF research. Oxidative stress and immune cells play a vital role in the pathogenesis of IPF. PON2 and TLR4, as oxidative stress-related genes, not only exhibit high diagnostic efficacy but also show close associations with immune cells. These findings suggest the potential of PON2 and TLR4 as novel diagnostic and therapeutic targets for IPF.

## Data availability statement

The datasets presented in this study can be found in online repositories. The names of the repository/repositories and accession number(s) can be found in the article material.

## Author contributions

CL: Conceptualization, Data curation, Formal analysis, Investigation, Software, Writing – original draft, Writing – review & editing. QA: Data curation, Supervision, Writing – review & editing. YJ: Methodology, Writing – review & editing. ZJ: Software, Writing – review & editing. ML: Funding acquisition, Writing – review & editing. XW: Funding acquisition, Writing – review & editing. HD: Supervision, Writing – original draft, Writing – review & editing.

## Footnotes

^1^ http://string-db.org
