# Corrigendum: Identification of oxidative stress-related diagnostic markers and immune infiltration features for idiopathic pulmonary fibrosis by bibliometrics and bioinformatics

**DOI:** 10.3389/fmed.2024.1503195

**Published:** 2024-10-16

**Authors:** Chang Li, Qing An, Yi Jin, Zefei Jiang, Meihe Li, Xiaoling Wu, Huimin Dang

**Affiliations:** ^1^Department of Traditional Chinese Medicine, The Second Affiliated Hospital of Xi'an Jiaotong University, Xi'an, China; ^2^Graduate School, Shaanxi University of Chinese Medicine, Xianyang, China; ^3^Acupuncture and Tuina School, Chengdu University of Traditional Chinese Medicine, Chengdu, China; ^4^Department of Renal Transplantation, The First Affiliated Hospital of Xi'an Jiaotong University, Xi'an, China; ^5^Department of Obstetrics and Gynecology, The Second Affiliated Hospital of Xi'an Jiaotong University, Xi'an, China

**Keywords:** oxidative stress, immune infiltration, idiopathic pulmonary fibrosis, bibliometrics, bioinformatics

In the published article, there was an error regarding the affiliation(s) for Chang Li. As well as having affiliation(s) [1], they should also have “Department of Traditional Chinese Medicine, The Second Affiliated Hospital of Xi'an Jiaotong University, Xi'an, China,” and the latter affiliation is ranked first.

In the published article, there was an error in the article type. Instead of “Review,” the article type should be “Original Research.”

Due to the article type change, a Data availability statement should be added for this article. The Data availability statement appears below.

## Data Availability

The datasets presented in this study can be found in online repositories. The names of the repository/repositories and accession number(s) can be found in the article material. The authors apologize for these errors and state that this does not change the scientific conclusions of the article in any way. The original article has been updated.

